# Abiotic Stress Tolerance in Plants: Brassinosteroids Navigate Competently

**DOI:** 10.3390/ijms232314577

**Published:** 2022-11-23

**Authors:** Abira Chaudhuri, Koushik Halder, Malik Z. Abdin, Manoj Majee, Asis Datta

**Affiliations:** 1 National Institute of Plant Genome Research, Aruna Asaf Ali Marg, New Delhi 110067, India; 2 Centre for Transgenic Plant Development, Department of Biotechnology, School of Chemical and Life Sciences, Jamia Hamdard, New Delhi 110062, India

**Keywords:** brassinosteroid, abiotic stress, signaling, transcription factors

## Abstract

Brassinosteroid hormones (BRs) multitask to smoothly regulate a broad spectrum of vital physiological processes in plants, such as cell division, cell expansion, differentiation, seed germination, xylem differentiation, reproductive development and light responses (photomorphogenesis and skotomorphogenesis). Their importance is inferred when visible abnormalities arise in plant phenotypes due to suboptimal or supraoptimal hormone levels. This group of steroidal hormones are major growth regulators, having pleiotropic effects and conferring abiotic stress resistance to plants. Numerous abiotic stresses are the cause of significant loss in agricultural yield globally. However, plants are well equipped with efficient stress combat machinery. Scavenging reactive oxygen species (ROS) is a unique mechanism to combat the deleterious effects of abiotic stresses. In light of numerous reports in the past two decades, the complex BR signaling under different stress conditions (drought, salinity, extreme temperatures and heavy metals/metalloids) that drastically hinders the normal metabolism of plants is gradually being untangled and revealed. Thus, crop improvement has substantial potential by tailoring either the brassinosteroid signaling, biosynthesis pathway or perception. This review aims to explore and dissect the actual mission of BRs in signaling cascades and summarize their positive role with respect to abiotic stress tolerance.

## 1. Introduction

Plants are sessile organisms and are invariably exposed to a plethora of environmental stress factors, both biotic and abiotic, the rapidly changing climatic conditions being a leading cause. Primarily, heat, cold, drought, flood, salinity and heavy metals/metalloids have posed potential threats to agriculture through generations, as they hamper the basic life mechanisms, both at a physiological and molecular level [1,2]. Numerous phytohormones interact with various factors such as reactive oxygen species (ROS), metabolites, etc., to expedite the plant’s response to any stressful stimuli [3,4]. Lately, BRs have taken a center stage in the phytohormone family, since they have displayed diverse roles in plant growth and development [5,6].

**Figure 1:**
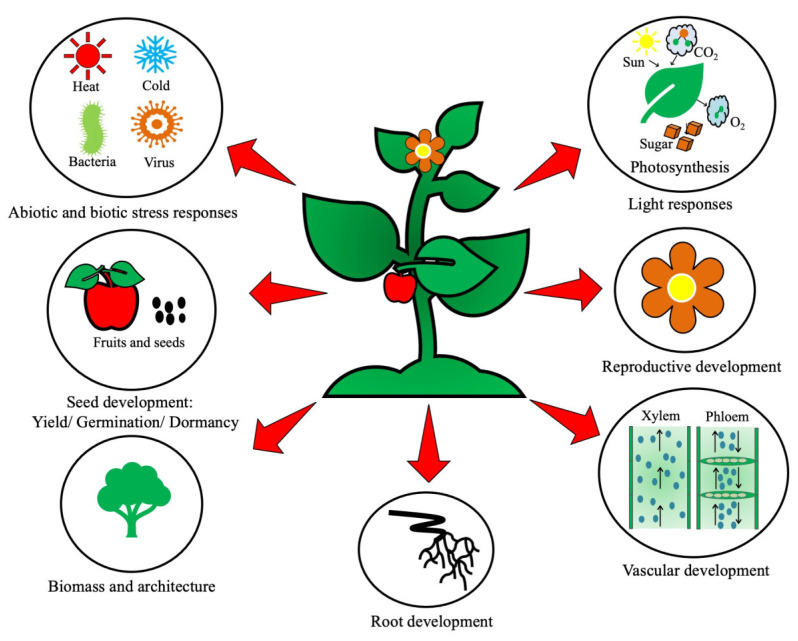
Active regulation of a wide array of developmental processes in plants by Brassinosteroids (BRs).

Rigorous research over the past few decades has proved with numerous supporting pieces of evidence that BRs possess significant power in mitigating the stress impact when plants are exposed to the above categories of stresses [7]. The plant-specific ligands of BRs directly bind to the cell surface receptors called leucine-rich repeat receptor kinases BRASSINOSTEROID-INSENSITIVE 1 (BRI1) and BRI1-associated receptor kinase (BAK1). The signaling happens via phosphorylation triggered inside the cytoplasm, involving phosphorylation of the BSU1 protein and the proteasome-mediated pulling down of BRASSINOSTEROID-INSENSITIVE 2 (BIN2) proteins. This deactivation of BIN2 paves the way for BRI1 EMS SUPPRESSOR/Brassinozole-resistant 1 (BES1/BZR1) to gain access to the target genes inside the nucleus [8]. This signaling cascade involves cross-talk with multiple phytohormones which have been duly established [9,10,11]. BRs participate in an active manner in plants to regulate diverse developmental processes [5], and a graphical representation is provided in Figure 1. Physiological abnormalities triggered by stress can be overcome by the exogenous application of BRs, and this phenomenon has gained considerable importance among scientists [12,13]. Research is continuing at the physiological and molecular level in different laboratories worldwide, yet the role of BRs in nullifying the adverse effects of stress is still an enigma. The reason for this haze in the signaling network is assumed to be not only the presence but also the cross-talk among them to eventually mitigate stress [14,15]. Here, we discuss in detail some of the potential abiotic stress factors and how their deleterious effects are modulated by BRs. The inferences gained here might be extrapolated for stress management in the case of many important food and cash crops. 

## 2. Brassinosteroid Signaling Cascade

Cell signaling via the cell-surface embedded receptors are the key to the survival and development of plants. In plants, signaling via cell surface receptors take place through ‘Receptor-Like-Kinases’ (RLKs) and they are considered to be the major type of receptors [16]. The structure of the RLKs is quite simple, consisting of three domains: an extracellular domain whose function is to attach to ligands, one transmembrane domain, and finally a cytoplasmic kinase domain which triggers all intracellular signal transmission. RLKs are the key participants of the signaling cascades that act during the defense responses in plants [17]. In case of BRs, the RLK which acts as the potential receptor for this steroid hormone is BRASSINOSTEROID INSENSITIVE 1 (BRI1). This receptor is the key regulator for a broad range of physiological and developmental processes in plants. Any type of mutation of BRI1 might have far-reaching negative consequences in plant development. Advanced research has identified many additional components of the BR signaling cascade, e.g., the co-receptor BRI1-Associated Receptor Kinase 1 (BAK1), GSK-3 like kinase BRASSINOSTEROID-INSENSITIVE 2 (BIN2), Bri1-suppressor 1 phosphatase (BSU1) and the Brassinozole-resistant (BZR) family of transcription factors [18]. 

Some very significant proteomic studies have also given helpful insights to researchers, as they established BR signaling kinases (BSKs) to act as substrate for the BRI1 kinase that acts as a connecting link between the receptor kinases present on the plasma membrane and the downstream cytoplasmic elements [19,20]. Further phosphoproteomic studies, especially detailed mass-spectrometry analysis, clearly established the presence and involvement of phosphorylation sites in the BR signaling cascade [21,22,23]. The BR signaling cascade can be divided into a stepwise process, as outlined below.

### 2.1. Jump-Start of BRI1 Receptor Kinase by Brassinosteroid

The BRI1 protein belongs to the leucine-rich repeat RLK (LRR-RLK) family. BRI1 is constructed of an extracellular domain consisting of 24 LRRs and a small island (ID) juxtaposed in between LRR20 and 21. BRs attach directly to the ID domain (ID-LRR21) which leads to the activation of the BRI1 kinase [24], which performs its assigned task of phosphorylating target proteins located in the cytoplasm and thus commences intracellular signal transmission. A plethora of mechanisms are interconnected in the whole process of ligand-triggered activation of BRI1. BRI1 kinase remains in a dormant state in the absence of BRs due to two partial hindrances: one being the unphosphorylated condition of the CT domain [25] and the other being attachment to the inhibitory protein BKI1 [26]. The attachment of BRs instantly triggers a series of molecular events that eventually leads to kinase activation. These molecular events include homodimerization and autophosphorylation of BRI1 [25,27], uncoupling of the BRI1 Kinase Inhibitor [26] and finally the attachment/transphosphorylation with the BAK1 co-receptor kinase [22,23]. 

### 2.2. Interaction of BRI1 with Receptor Complex Associates 

BAK1 is an RLK that interplays with BRI1. BRI1 kinase is the key player that is kicked into action the moment BRs come into the scenario, linking BRI1 with BAK1, which can be vouched by numerous evidence. Firstly, it was proved that mutant BRI1 sans kinase activity betrayed a remarkable decline in binding with BAK1 both during in vitro and in vivo conditions. However, in case of mutated BAK1 kinase there was only a negligible decline in interaction [28,29]. The second piece of evidence is that BR is capable of triggering linkage between a wild-type BRI1 with a ‘kinase-dead’ mutant BAK1 but not vice versa [22]. In another line of evidence, it was seen that BR fails to hike up the phosphorylation of BAK1 in the *bri1-1* mutant but can perform the exact opposite during BRI1 phosphorylation in the *bak1*, *bakk1* double mutant. The observation and inferences obtained from the in vitro kinase assays gave a picture that BAK1 actively transphosphorylates BRI1, which bestows extreme power to BRI1 kinase activity towards its substrate peptide. Therefore, to sum up it can be stated that the phosphorylation of BAK1, which is totally BR induced, is controlled by BRI1 kinase activity, whereas in another line of action, the transphosphorylation of the substrate peptide by BRI1 is severely boosted by phosphorylation of BAK1 [22]. However, studies by two prominent research groups [28,29] paved the way to concluding that the overexpression of a dominant negative mutant *bak1* resulted in a significant dwarf phenotype, and this points clearly to that the presence of BAK1 is mandatory in the BR signaling cascade. 

### 2.3. Joining the Dots to Complete the BR Signaling Cascade

The downstream components of the BR signaling cascade are numerous and significant. To start, there is BIN2, which is a GSK3/SHAGGY-like protein kinase; BSU1, a phosphate with Kelch repeats; and finally, the transcription factors BZR1 and BZR2, which are also called by the name BES1 [16]. When BR is not present in the scene, the BR signaling cascade faces a negative command by BIN2, which phosphorylates BZR1 and BES1/BZR2 to carry out this hindrance [30,31]. BZR1 and BES1/BZR2 are disabled from functioning due to phosphorylation by BIN2, and this process takes place in diverse ways: the first being by hindering DNA binding and nuclear localization and the second being by advocating degradation by the proteasome [30,31,32,33,34]. The S173 residue of BZR1 is phosphorylated and this leads to a potential association with the 14-3-3 proteins, which transport and maintain BZR1 and BZR2/BES1 inside the cytoplasm [33,34]. The exact opposite activity of BIN2 is performed by BSU1 phosphatase, which facilitates dephosphorylation of BES1/BZR2 in plants [35].

**Figure 2:**
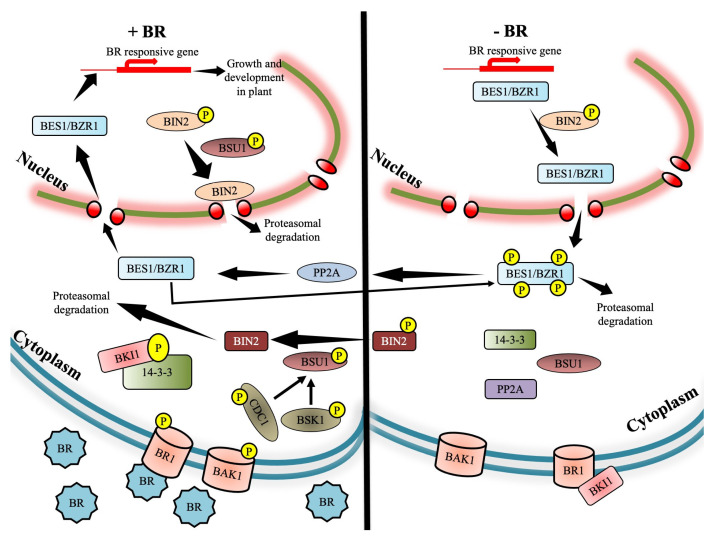
A comprehensive diagram depicting the brassinosteroid signaling pathway in plants.

Many research groups were in a dilemma in the past about the exact function of BSU1, which was presumed to participate in the dephosphorylation process of BES1/BZR2. However, extensive research [20] has shed light on the biochemical and genetic aspects of the BR signaling cascade. This has given a vivid picture that BSU1 has a direct downstream location from BSK1 and upstream of BIN2. BSU1 functions in a very interesting way by disabling BIN2, rather than directly dephosphorylating BES1/BZR2 or BZR1, which in turn stops phosphorylation, and this disabling happens by dephosphorylation of a phospho-tyrosine residue (pY200) of BIN2. Once again, [20] pinpointed that BSU11 has some versatile traits, such as interacting with BIN2 as well as BSK1 directly, both in vitro and in vivo. BSU1 strongly binds with BSK1, and the condition for this happens to be BSK1 being phosphorylated by BRI1 and almost annihilated by a mutation at the BRI1 phosphorylation site (S230A). The activation of BSU1 happens by phosphorylation, and this is carried out by BSK1. In addition, was inferred that BR treatment causes a spike in BSU1′s inhibiting capacity of BIN2. Finally, we can draw a conclusion about the complete BR signaling pathway, which comprises a line of chronological events starting from the kick-start of BRI1, BSK1 and BSU1, deactivation of BIN2 and accretion of unphosphorylated BZR1 along with BZR2/BES1 inside the nucleus. This entire mechanism deciphered by research provides a concrete basis of a ligand’s grasp by RLK and links it with the downstream transcription factors in a plant body. However, one missing link still remains to be found, and that is the identity crisis of the protein phosphatase responsible for the dephosphorylation of the BZR transcription factor. Further genetic studies using mutants clearly point out that the pathway linking BRI1 to BZR1/BES1 is the main signaling route for the majority of BR-induced responses. A summary of the BR signaling pathway is shown in Figure 2.

## 3. Signaling and Regulation by BRs (Endogenous and Exogenous) in Plants under Abiotic Stress

**Figure 3:**
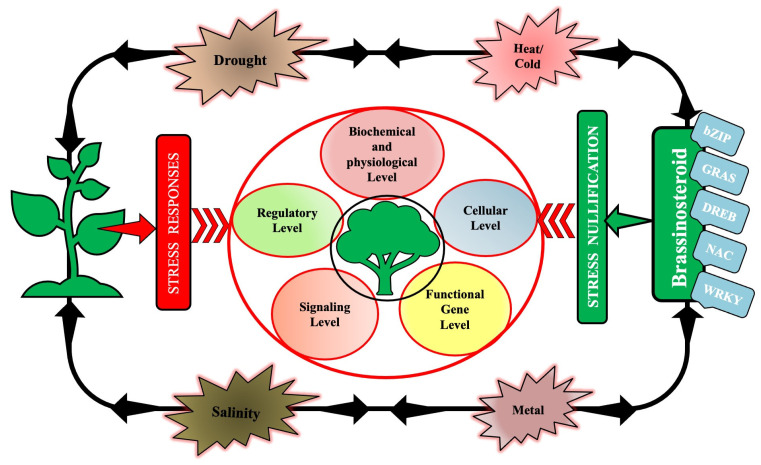
A vivid picture of the numerous mechanisms endorsed by the Brassinosteroids at different levels in plants to mitigate a plethora of potentially fatal abiotic stresses with close association with transcription factors.

It has already been established by various research groups that BRs control some crucial physiological and biochemical processes such as cell differentiation, cell division, elongation, etc. The model plant *Arabidopsis thaliana* is a potential platform for experimentation on BRs and has been a source of many recent findings. Plants have a labyrinth of extraordinary signaling networks that sense and respond appropriately to the ever-changing environment. In the present day, the nature of stress is also rather complex, therefore the active participation of a multitude of sensors for signal perception and transmission are preempted. The signaling cascades are activated spontaneously after the stress trigger, which eventually activates the stress-responsive genes. According to established results, the membrane-based steroid receptor BRI1 binds with BR, and this triggers a chain reaction of cytoplasmic signaling that leads to the expression of BR-associated genes. It was well observed in this context that in *Arabidopsis thaliana* the primary roots have shown precise stress-responsive signals as an adaptive response to stress [10]. More and more scientists are working presently on BR-associated stress-responsive signaling mechanisms which are potential routes to decode the plant’s adjusting capacity to biotic and abiotic stress [36,37]. BRs balance environmental assaults and the normal growth process by operating through several signaling routes, e.g., acting independently by engaging in cross-talk with other phytohormones [10]. The machineries that are activated by BR signaling to bring about adaptive response are the elevated production of osmoprotectants [38], the triggering of antioxidant production machinery [39] and the arousal of stress-responsive transcription factors [40]. BRs have a considerably significant interaction with a variety of stress-related transcription factors (TFs), directly or indirectly. This operation takes place through the pathway involving the negative regulator BIN2 and prominent TFs BZR1/BES1 that eventually trigger stress-adaptive signaling pathways. Other potential transcription factors that take part in this entire operation of synchronizing abiotic stress response are DREB, WRKY, MYB/MYC, GRAS, bZIP, NAC, NPR, etc. [41]. Figure 3 gives a graphic detail of the mechanisms adopted by BRs in positively controlling abiotic stresses in plants through signaling at various levels by actively interacting with TFs. 

Ref. [42] showed that BRs are associated with nitrogen (N) starvation in plants and the responses are via the modulation of autophagy, which is a self-destructive process as known to all and followed by plants to mediate stress response. Next comes the entry of the exogenous application of BRs. Exogenous BRs elevate the transcript levels of autophagy-associated genes and the generation of autophagosomes [43]. Various pharmacological approaches give us an idea about the effects of exogenous application of BRs on plants to infer the stress-responsive/stress-protective role of BRs. Different pharmacological techniques of application such as foliar spray, pre-sowing seed treatment, pre-planting, dipping of cuttings, post-emergence root treatment, etc., have been applied to a vast range of plant species [44,45,46,47,48]. Many scientist groups [47,49] have established that the effects of exogenously applied BRs depend on quite a number of parameters, such as plants, dose, growth stage, conditions of growth, viz., with/without stress, signaling molecules, growth regulators, cross-talk with other hormones, etc. 

In the forthcoming section we discuss in detail the effects of BRs (endogenous and exogenous) that contribute to abiotic stress tolerance in plants, how BRs function in modulating the responses of plants to numerous deleterious stress factors such as heat, cold, drought, salinity and heavy metals, and the procedure and working of the BR-mediated escalated tolerance level of plants to abiotic stress factors. 

### 3.1. BRs in Mitigating Heat and Cold Stress

#### 3.1.1. Heat Stress

Global warming has proved to be an extremely deleterious stress factor in the field of agriculture and plant growth [49]. High temperature causes damages such as leaf burn, stunted plant growth (root and shoot), abscission and senescence, fruit burns, etc., which lead to reduced crop (food and cash) productivity. Dissecting the stress-related phonotypic changes, when studied at the molecular level, it was observed that high temperatures trigger the aggregation of BES1 and BZR1, which leads to high levels of PHYTOCHROME INTERACTING FACTOR4 (PIF4) [50]. Subsequent changes that happen are the formation of heterodimers of PIF4-BES1 that ease the action of BZR1 on gene transcription, eventually directing the plants towards thermogenic growth. On the contrary, low BRI1 levels under the influence of high temperatures have a profound effect on BR signaling, due to which there is a drastic increase in root growth [51]. 

Apart from stress-mitigating functions of the endogenous BR signaling cascade, exogenously applied BR also has the same functions in plants [52]. Among the physiological processes which are extremely sensitive to heat stress, photosynthesis occupies the topmost position [53]. It was proven some time ago by scientists that extreme high temperatures hinder the efficiency of photosystem II (PSII) and drastically and simultaneously cause a dip in the net photosynthetic rate [54,55]. Some potential examples can be cited in the case of tomato, where a pretreatment with epibrassinolide (EBR) can reverse the above-discussed adverse side effects caused by high temperature by elevating the levels of antioxidant enzymes that lower subsequent lipid peroxidation in such stressful conditions [54]. BRs have proven to multitask, as they help the genotypes of plants (thermotolerant and thermosensitive) to improve their thermo-tolerance, which can be seen in the case of heat-sensitive and heat-tolerant ecotypes of melon under extreme high temperature. In that study, a pretreatment with EBR enhanced the contents of photosynthetic pigments to a great extent, and other parameters such as net carbon dioxide assimilation rate, photochemical activity of PSI, stomatal conductance, water-use efficiency, etc. [55]. In another case with eggplant, exogenous EBR treatment nullified the adverse effects of heat stress by modulating the aggregation of reactive oxygen species (ROS) by extreme heat [56]. Foliar application of EBR in wheat under very high temperatures enriches biomass accumulation, growth, photosynthetic efficiency and last but not least the antioxidant potential [57]. An interesting phenomenon has been noticed in heat-stressed rice by [58], where the exogenous application of BRs mimics a compound called 7,8-Dihydro-8œ-20-hydroxyecdysone (œDHECD). These changes happen in the reproductive stage and eventually spike up photosynthesis, carbohydrate load, seed setting and seed weight. Clearly now a conclusion can be established relying on the above examples that BRs act as a potential tonic for plant photosynthesis and antioxidant function, finally leading to alleviation of the hazardous effects of high temperatures. 

Molecular genetic studies offer us a handful of evidence which buttress the in-depth mechanisms adopted by BRs in mitigating heat stress. Reference [59] gave a clear picture in tomato, in that a transient apoplastic production of H_2_O_2_ (NADPH oxidase dependent) is the biochemical cue to kick-start the BR signaling cascade, which will ultimately safeguard the plant from the adverse effects of heat stress. It was also deduced from experimentations that exogenous BR upregulates the transcript levels of stress- and defense-associated genes (e.g., *APX5*, *WRKY1*, *PRI*, *NPR1*, *GR1*, *CAT1*, *Cu-Zn SOD* and *HSP90*) in tomato, thus proving once again the function and importance of BRs in stress resistance/tolerance. Along with the antioxidant machinery of the cell, heat-shock proteins (HSPs) hold a major position in this entire BR signaling cascade, leading to thermotolerance. Exogenous application of EBR increases HSP biosynthesis during prolonged heat stress by acting as a protective shield for various members of the translational apparatus [60]. Some contradictory reports have popped up from *Arabidopsis* research, where experimentations [61] give a clear hint that HSP aggregation is not at all mandatory for BR-triggered thermotolerance. Overexpression of *AtDWF4* (BR biosynthesis-related gene) has no effect in mitigating the adverse effects of stress, both heat and salinity, whereas in the case of barley the mutants (BR deficient, impaired in signaling) show a miraculous phenotype of being heat stress tolerant compared to wild-type varieties [62]. Finally, it can be concluded that the signaling cascades adopted by endogenous BRs and exogenous BRs are varied in case of various plants [63]. 

#### 3.1.2. Cold Stress

Various parts of the world are inhabited by thermophilic plants, and for them, extreme low temperatures induce freezing or chilling stress and paralyze their life processes majorly [64]. In addition, along with various changes in cell architecture and osmotic properties, cold stress severely hampers the carbon dioxide assimilation rate, and causes photoinhibition of the PSI and the PSII photosystem along with reduced enzyme activity of the Benson–Calvin cycle [64]. It is well established by various scientists globally that any type of stress triggers ROS accumulation, and large quantities of ROS can have severe deleterious effects especially to bio-membranes by causing lipid peroxidation [65]. To combat cold stress and accumulation of threatening amounts of ROS, plants have developed a wide range of processes and procedures of scavenging them [66]. It has been established [67] that by overexpressing the genes of the ROS scavenging machinery, the plants can be better equipped to tolerate chilling stress. It was shown that plants deficient in BR have reduced capacity to chilling tolerance because of various alterations in cellular, biochemical and molecular properties. The application of exogenous EBR to a tomato plant or the overexpression of the *DWRF* gene causes a rise in the plant’s capacity to tolerate cold stress and reduces the damage caused by ROS considerably [68]. During cold stress tolerance, ROS act as mediator signal molecules in the BR orchestrated signaling cascade in the case of tomato. EBR applied exogenously has certain beneficial effects, such as revamping CO_2_ assimilation and relieving photoinhibition of PSII caused by cold stress. The exogenous EBR treatment revives the plant’s photosynthetic machinery by boosting up the enzymes of the ROS scavenging system (ascorbate–glutathione cycle, AsA-GSH) and other machineries to maintain oxidation–reduction balance in the cells [69]. Some notable examples can be cited here, such as in case of *Vitis vinifera* (grapes) seedlings where exogenous EBR treatment attunes the AsA-GSH signaling cascade, transiently facilitating chilling tolerance [70]. Foliar spray of EBR minimizes the adverse effects of chilling in grapevines. It minimizes the lipid peroxidation of the membranes by modulating the antioxidative potential [71]. 

To combat cold stress, endogenous BRs play an active role in surpassing photoinhibition, and the technique adapted by the plants is ‘photoprotection’. When the ambient conditions are stressful the plants gather active BRs, which consequently triggers the activation of BZR1, and this action subsequently leads to spike up the *RESPIRATORY BURST OXIDASE HOMOLOG 1* (*RBOH1*) transcript levels following generation of high levels of apoplastic H_2_O_2_ [72]. In contrast, just the opposite scenario happens in the case of a mutation in *BZR1* or suppression of *RBOH1*. This annuls the photoprotection offered by BRs and renders the plant prone to chilling-induced photoinhibition. C-REPEAT/DEHYDRATION-RESPONSIVE ELEMENT BINDING FACTOR1 (CBF1) plays a major role in BR-dependent chilling stress tolerance in plants. BRs uplift the freezing tolerance capacity of plants via CBF-1 dependent or independent signaling cascade, and this is mediated via the arousal of *COR*-*RESPONSIVE* (*COR*) genes [73]. To enhance cold tolerance, the BR signaling pathway allows huge aggregation of BZR1 and BES1 in their active unphosphorylated state that encourage the transcription of *CBF1* and *CBF2* to facilitate chilling tolerance [74]. However, in another report [75] it was clearly stated with supporting evidence that BRs negatively control cold stress response during long episodes of chilling phase, when BIN2 damages the transcription factor INDUCER OF CBF EXPRESSION1 (ICE1). From the above analysis it can be inferred and concluded that BRs have two potential functions [49]: one is to foster stress tolerance, and another is to minimize stress response, and both of these functions majorly rely on spatiotemporal management. 

#### 3.1.3. Drought Stress 

Drought, a major setback to agriculture in the tropical countries, has caused severe economic loss to countries’ economies and is a major threat to food security. Lack of rainfall or proper irrigation facilities are the primary reasons for drought stress, which eventually leads to osmotic stress. Exposure to osmotic stress can prove extremely deleterious to plants at molecular and cellular levels and to the plant as a whole. There can be developmental and morphological changes, such as inhibition of shoot growth and escalation of root growth, modulation of ion transport, alteration in metabolism and ultimately disturbing of the plant’s homeostasis [76]. Drought triggers the heavy secretion of the phytohormone abscisic acid (ABA). The authors of [77] studied this in detail and showed that exogenous application of BRs can magnify the ABA content in the cell and save plants from the adverse effects of drought. Almost a decade ago, [78] performed EBR treatment on tomato, subjected it to stress conditions, and inferred that this procedure enhances photosynthetic capacity, antioxidant defense machinery and leaf water conditions, which have beneficial outcomes in combating stress. Similar experiments were performed [79] by applying foliar spray of BRs on pepper, which had a positive effect on the efficiency of light application and implementation along with the dispersal of the excitation energy through the PSII machinery during severe drought. Studies [61] reveal that exogenous application of BR modulates the gene expression pattern of those genes that participate in the coding of structural and regulatory proteins. As seen in the case of *Brassica napus*, exogenous EBR caused a hike in transcription of two potential drought-responsive genes (*BnCBF5* and *BnDREB*), and this overall change had a partial if not total drought-tolerant effect on the plant seedlings. Some experiments have shed light on how an exogenous treatment of BRs can mollify the deleterious effects of long-term stress on plants. As in the case of *Brassica juncea*, the picture was clear that prolonged drought stress for 7 days arrested its growth and photosynthesis capacity, but a course of 26-homobrassinolode (HBL) treatment even after a month from sowing can annul all the negative side effects mentioned before [80]. ROS accumulation is an integral side effect of stress of any type. In another study in the case of drought stress, heavy aggregation of ROS was noticed, and once again BR treatment came to the rescue. It significantly brought a dip in the ROS levels and consequent lipid peroxidation of the drought-exposed membranes [81]. Exogenous treatment with BR has proved to be a great boon to the agriculture sector for improving the plants’ capacity to tolerate drought stress. Recent reports show that a foliar spray of a BR analogue DI-31 showed remarkable beneficial effects on various physical and biochemical parameters in Lulo plant (*Solanum quitoense* L.cv. *septentrionale*) seedlings, which were subjected to drought stress followed by foliar spray of a BR analogue (DI-31). The positive effects noticed were elevated photosynthetic efficiency of the PSII photosystem, enhanced rate of plant growth, elevated concentration of photosynthetic pigments and minimal lipid peroxidation of the membranes. These results paved the way for DI-31 to be globally used as a tool for water stress management in moderately to poorly irrigated (naturally or artificially) areas [82]. A group of scientists [49,63,83] have shown that plants treated with exogenous BRs are no doubt drought tolerant, but at the same time both BR-deficient and BR-insensitive mutants too have enhanced capacity to tolerate stress. In the case of tomato, it has been clearly proved that a rise in the level of BR biosynthesis and not the BR perception and signaling is actually the key player that enhances the tolerance capacity. At the same time, overexpression of the *BRI1* gene has a negative impact on the drought tolerance property of tomato, which clearly indicates that complex loopholes exist in the BR signaling cascade that might tune the stress tolerance capacity of plants either way [49,63]. 

#### 3.1.4. Salinity Stress

Salinity imposes severe osmotic stress on plants growing on saline soil which results in plasmolysis of cells, and this leads to the condition called physiological drought. This situation is an extreme deleterious condition hampering plant growth and development, eventually leading to poor crop yield. The effect of exogenous BRs has been studied in a variety of plants, including the model plant *Arabidopsis thaliana* and others such as *Brassica napus* (mustard), *Solanum melongena* (eggplant), *Capsicum annuum* (pepper), *Zea mays* (maize), *Robinia pseudoacacia* L. (black locust) and *Phaseolus vulgaris* (common bean) [48,78,84]. Eggplants treated with exogenous BRs show an increased tolerance to saline stress, and this observation is supported by numerous evidence such as enhanced activity of antioxidant enzyme scavenging the deleterious free radicals and decreased concentrations of Na^+^ and Cl^−^ ions, along with an increase in the concentrations of the essential metal ions K^+^ and Ca^2+^ [78]. A foliar spray of HBL is an effective antidote against salinity stress in rapeseed, and this works up to a span of 45 days after sowing [85]. In cucumber plants, application of EBR under salt stress lowers the concentrations of NH_4_^+^ and NO_3_^−^ ions significantly, and the consequent enhancement in photosynthesis, efficient utilization of nitrogen and total polyamine content can be attributed to this enhanced tolerance [78]. Black locust combats stress by following almost the same strategy as the previously discussed cases. Here, the external application of EBR enhances the major life processes such as the maximum quantum efficiency of PSII, the net photosynthetic rate, transpiration rate, stomatal conductance and chlorophyll content [48]. Sometimes BRs can multitask and mitigate the stress effects of more than one stress factor, when applied on plants. Potential examples of this are EBR mitigating the combined salt stress imposed on mustard by Sodium Chloride (NaCl), Nickel Chloride (NiCl_2_) and HBR, shown to alleviate stress conditions induced by both salt and heat in mung bean [84,86]. 

Ubiquitin-conjugating enzyme 32(UBC32) plays a vital role in BR-mediated/induced tolerance to salt stress in plants. Since it is a major functional element of the endoplasmic-reticulum-associated protein degradation (ERAD) signaling cascade, UBC32 triggers the aggregation of the BRI1 receptor inside the cells and tunes the ERAD signaling cascade to act in rhythm with the BR signaling cascade, which enhances salt tolerance in the model plant *Arabidopsis thaliana*. In a study by [44], it was established that BRs have some role in the epigenetic modification of DNA in plants under salinity stress. It has been pointed out that BRs modulate DNA methylation, which is majorly responsible for any plant’s salt tolerance property. A case has been cited where seed priming with EBR begot complete methylation and enhanced salt tolerance in plants. 

#### 3.1.5. Heavy Metal Stress

The Industrial Revolution has proved to be a boon and a bane at the same time to society. As a global boon, life has become much easier with urbanization and modernization, but the bane looms up menacingly over the skies with fossil fuel combustion and pollution caused by various heavy metals/metalloids. These have proved to be potential health hazards for humans, animals and plants [87,88]. All the food crops and useful plants that grow in this heavy metal/metalloid-polluted soil are severely stressed with heavy poisonous metal loads in their systems. This has been considered by different groups of scientists as a unique type of stress, since the plants are weak and heavily compromised in quality. Additionally, the metals build up in magnitude through the food chain and pose as poisonous to humans and animals [89,90]. To bring out a substantial way out from this heavy metal pollution and stress, various approaches have been attempted by researchers. The signaling pathways of endogenous phytohormones have been modulated, exogenous plant growth regulators (BRs) have been put to use and bioactive compounds have been tried out; all these endeavors have shown promising prospects to combat heavy metal stress [91,92]. 

Heavy metals/metalloids have extreme adverse effects on CO_2_ assimilation potential and the photosynthetic machinery of plants [93]. The example of Cadmium (Cd) can be cited, where Cd poisoning in plants affects the photosynthetic rate by lowering it drastically. The Calvin cycle is directly affected by Cd, which hinders the usage of ATP and NADPH. Cd stress in tomato results in a drastic decrease [94] of the stomatal conductance, photosynthetic rate, maximal quantum efficiency of PSII (Fv/Fm, Ø_PSII_) and the photochemical quenching coefficient (qP). The fall in the carbon assimilation competency is directly proportional to the photosynthetic pigment content and inversely with the Cd aggregation in the leaves. Therefore, the increase in plant biomass becomes practically impossible in stressed plants. Such a condition can be bypassed even in stressed plants by a foliar spray of EBR. This improves the photosynthetic pigment content, CO_2_ assimilation capacity and Fv/Fm, and simultaneously hinders Cd uptake via roots and its translocation upwards towards stem and leaf, eventually increasing the plant biomass. Similar to EBR, HBL can also perform similar functions in mitigating Cd-triggered stress in tomato [95]. A commendable improvement in fruit yield has been observed by a foliar spray of BRs (both EBR and HBL) for tomato plants even in heavily Cd-contaminated soil [96]. BRs are extremely efficient in giving results and that too within a short span of time, and this can be seen in a case where a single foliar dose, approximately a day before assessment, caused a drastic rise in the photosynthesis capacity in tomato even after the plant was exposed to almost 2 months of Cd stress [97]. In the case of *Cicer arietinum*, HBL acts effectively in alleviating the Cd-triggered phytotoxicity by elevating the levels of antioxidant enzymes as well as other effective cellular ROS scavengers [98]. 

Tobacco leaf mesophyll cells adopt a strange appearance under Chromium (Cr) stress. Electron micrograph pictures have revealed majorly distorted cell wall and cell membrane along with dilated thylakoids. It was EBR application that relived the plant and protected its organelles, especially the chloroplast along with the organization of grana and thylakoids [99]. Under any type of stress, heavy metal in this case, ROS production is incited, and this brings about deleterious effects on plant metabolism causing excessive injury to nucleic acids, proteins and lipids [100]. Apart from causing extreme harm to plants, some heavy metals such as Nickel (Ni) have the potential to boost the biosynthesis of a range of BRs (epibrassinolide, dolicholide, castesterone and Typhasterol), and this has been observed in *Brassica juncea* [101]. In legumes such as *Vigna radiata*, the exact opposite effect of Ni stress has been noticed, where EBR comes to rescue and promotes nodulation, and this consequently benefits the overall growth of the plant. Zinc oxide nanoparticle induced stress has been studied by scientists where EBR dissolved in half-strength MS medium rescued the tomato seedlings, as it boosted the antioxidant enzymes for rapid scavenging of ROS [102]. In summary, BRs work effectively in the amelioration of heavy metal stress in plants by substantially increasing the photosynthetic pigment assemblage and carbon metabolism and the antioxidant defense machinery (including both the enzymatic and non-enzymatic components), along with their ROS scavenging efficiency and antioxidant content such as glutathione and phytochelatins [103]. Up to now, very little information has been obtained on how the BR signaling cascade behaves in plants under Zinc (Zn), Boron (Bo), Cobalt (Co), Manganese (Mn) and Arsenic (As) stress. In *Raphanus sativus* seedlings under Zn stress, external supplementation with EBR enhanced the activity of antioxidant enzymes, reinforced GSH metabolism and enhanced the assemblage of non-enzymatic antioxidants and proteins, which aided in their successful survival in Zinc toxicity [104]. EBR supplementation has been applied in the case of *Raphanus sativus* L. seedlings subjected to Pb toxicity. This treatment reduced the metal toxicity significantly by triggering CAT, APX, GPX and SOD activity and simultaneously decreasing POD activity [7]. In tomato plants under Pb stress, EBR once again triggered the hike in activity of the ROS scavengers, eventually mitigating the ill effects of stress [105]. 

HBL and EBR both were reported to be active in the case of *Brassica juncea* and *Zea mays* plants under Zn toxicity. The ROS scavenging enzymes, namely superoxide dismutase (SOD), catalase (CAT), peroxidase (POD), ascorbate peroxidase (APX), guaiacol peroxidase (GPX), glutathione reductase (GR), monodehydroascorbate reductase (MDHAR) and dehydroascorbate reductase (DHAR), as well as the non-enzymatic scavengers, namely GSH, minimize the metal poisoning triggered lipid peroxidation of the membranes [106,107]. When HBL was applied to Bo-exposed *Vigna radiata*, antioxidant enzyme (CAT, POD, SOD) activity was accelerated, and this caused a huge positive impact on the growth, photosynthesis and water relations [108]. EBR foliar application in *Brassica juncea* plants under Co stress showed significant tolerance, since the activities of SOD, CAT, POD, GR, APX, MDHAR and DHAR enzymes were increased noticeably [109]. Similarly, BR-mediated enhanced tolerance to As in *Raphanus sativus* was attributed to the increased activity of SOD and CAT [110]. When exposed to high levels of Mn, once again EBR comes to rescue in case of *Zea mays*. EBR enhanced the activities of the major antioxidant scavenging enzymes, which consequently blocked lipid peroxidation and quenched the harmful free radicals (superoxide and H_2_O_2_) [111]. Though various research groups have conducted in-depth analysis of the pathway which BR sticks to in quenching heavy metal stress, still no reports of a potential cross-talk between an endogenous BR signaling cascade being triggered by exogenously applied BR have arrived yet. 

## 4. Future Prospects

The present global climatic scenario is adverse and highly threatening, which has imposed a vast range of abiotic stresses on plants, ultimately limiting crop production. However, it has been proved that plants can cope with these stresses, yet the deteriorating climatic conditions and the increasing intensity of pressures have outpaced their potential for successful adaptation. Therefore, utilizing external resources to aid plant stress tolerance is imperative. A pertinent example is the Green Revolution, which drastically altered agriculture’s face. However, now, new climate issues have proved as limitations which have challenged the sustainability of the Green Revolution crop varieties. Some advanced tools and techniques can simplify many complexities in this study. The considerably new tools, precise translatomics [112] and florescent-activated cell sorting (FACS) [113], can efficiently map BR responses, which subsequently can trace the cross-talk of BRs with other phytohormones in a specific tissue under various stress stimuli. Highly advanced tools and techniques should be established to track the distribution of BRs and their activity under deleterious stress conditions. 

The exogenous application of BR is gaining immense importance, which regulates BR levels in crop plants bred during the Green Revolution and makes them resilient to numerous abiotic stress factors [114]. BRs can be vastly used in agriculture, especially cereal crops, to increase productivity and make them heat tolerant. A transgenic approach can prove highly fruitful, where genes associated with BR biosynthesis and signaling are targeted to generate transgenic cereal crops with increased productivity. The exogenous application of BRs at different abiotic stress conditions on various cereal crops can be tested to obtain a complete idea of their activity. The results from such experimentations can impart in-depth knowledge to the growers about which specific BR to use on which cereal variety, in what concentration, etc. Agronomy and molecular biology can be merged to create a complete network linking phenotypic alterations induced by BR treatment and the corresponding underlying molecular changes. It is of utmost importance to trace the exact transport route of exogenously applied BR and its longevity inside the cell, which will open numerous windows of study of abiotic stress tolerance. Furthermore, the impact of exogenous BR on the biosynthesis and signaling of other phytohormones under stress conditions is a promising area of research. Genetic engineering, which can manipulate BR genes along with the above-discussed tools and techniques, can boost agricultural yields with improved tolerance capacity to abiotic stresses. 

## 5. Conclusions

A lot of discussion worldwide has successfully proved that environmental stresses are the primary reasons for heavy losses in crop yields globally. As a reliable strategy to overcome stress and ensure survival, plants operate via a three-step route, which is (i) maintenance of cellular homeostasis, (ii) detoxification, and finally, (iii) recovery of the growth process. The phytohormone BR and various other related compounds have been reported to be effective counteracting antidotes to various kinds of abiotic stresses, including heat, chilling, drought, salinity, heavy metal, etc. BRs are considered very crucial in this case, since they mediate the stress tolerance responses by modulating a precise set of genes. BRs control the transcription of these vital genes, since they encode vital proteins and enzymes whose presence is mandatory to win the stress battle. BRs are generally not too active during normal growth conditions in plants, but they show their beneficial prowess in a stressful ambience. The beneficial activities brought about by BRs in mitigating the effects of stress are BR-induced ROS scavenging, maintaining redox homeostasis, improved carbon assimilation, photoprotection and improvement in antioxidant potential (enzymatic ROS scavengers and non-enzymatic ROS scavengers). Some other beneficial activities triggered by BRs during stress are improved defense response, detoxification potential, autophagy, etc. Extensive information has been gleaned from the research by various groups on this topic, yet much remains unchecked in the list. More and more investigations will provide insights into the still-arcane area of molecular–genetic operations of BRs and other related compounds, which modulate the antioxidant defense machinery and ultimately control the deleterious stress responses in plants.
